# Crosstalk between macrophages and cardiac cells after myocardial infarction

**DOI:** 10.1186/s12964-023-01105-4

**Published:** 2023-05-11

**Authors:** Yuhong Jian, Xiao Zhou, Wenju Shan, Cheng Chen, Wei Ge, Jun Cui, Wei Yi, Yang Sun

**Affiliations:** 1 Department of General Medicine, Xijing Hospital, The Fourth Military Medical University, Xi’an, China; 2 Department of Anesthesiology, Renji Hospital, Shanghai Jiao Tong University School of Medicine, Shanghai, China; 3 Department of Cardiovascular Surgery, Xijing Hospital, The Fourth Military Medical University, Xi’an, China

**Keywords:** Macrophages, Crosstalk, Myocardial infarction, Signaling pathway

## Abstract

Cardiovascular diseases, such as myocardial infarction (MI), are a leading cause of death worldwide. Acute MI (AMI) inflicts massive injury to the coronary microcirculation, causing large-scale cardiomyocyte death due to ischemia and hypoxia. Inflammatory cells such as monocytes and macrophages migrate to the damaged area to clear away dead cells post-MI. Macrophages are pleiotropic cells of the innate immune system, which play an essential role in the initial inflammatory response that occurs following MI, inducing subsequent damage and facilitating recovery. Besides their recognized role within the immune response, macrophages participate in crosstalk with other cells (including cardiomyocytes, fibroblasts, immune cells, and vascular endothelial cells) to coordinate post-MI processes within cardiac tissue. Macrophage-secreted exosomes have recently attracted increasing attention, which has led to a more elaborate understanding of macrophage function. Currently, the functional roles of macrophages in the microenvironment of the infarcted heart, particularly with regard to their interaction with surrounding cells, remain unclear. Understanding the specific mechanisms that mediate this crosstalk is essential in treating MI. In this review, we discuss the origin of macrophages, changes in their distribution post-MI, phenotypic and functional plasticity, as well as the specific signaling pathways involved, with a focus on the crosstalk with other cells in the heart. Thus, we provide a new perspective on the treatment of MI. Further in-depth research is required to elucidate the mechanisms underlying crosstalk between macrophages and other cells within cardiac tissue for the identification of potential therapeutic targets.

Video Abstract

**Supplementary Information:**

The online version contains supplementary material available at 10.1186/s12964-023-01105-4.

## Background

Acute myocardial infarction (AMI) and subsequent heart failure are among the leading causes of death and disability worldwide. Timely reperfusion therapy has revolutionized the emergency management of AMI. The most effective treatment following AMI includes immediate myocardial reperfusion using primary percutaneous coronary intervention and coronary artery bypass grafting. This reduces acute myocardial ischemia/reperfusion injury, preserving viable myocardium and limiting myocardial infarct size [1–3]. However, myocardial reperfusion may itself cause myocardial cell death and injury, also known as ‘myocardial reperfusion injury’, accounting for 50% of the final myocardial infarction (MI) area [4]. Therefore, AMI-associated mortality and morbidity remain significant, at 7% and 22%, respectively [5]. New therapies are needed to reduce infarct size and thus prevent adverse left ventricular remodeling. This would reduce the incidence of heart failure after AMI and improve clinical outcomes.

The innate immune response is an important regulator of the response to AMI, which consists of inflammatory, proliferative, and maturation phases [6, 7]. Monocytes and macrophages take center-stage in all three phases. The numbers of cardiac monocytes and macrophages increase rapidly after AMI [8, 9]. The initial populations of infiltrating monocytes and macrophages exhibit a pro-inflammatory phenotype which shifts to a predominantly anti-inflammatory phenotype over the following days, coordinating the deposition of scar tissue [10]. However, there is increasing evidence that several different cell types are involved in the inflammatory response. These together regulate AMI, which highlights their potential as therapeutic targets for cardiac recovery. Here, we discuss the origin and distribution of macrophages following MI. Next, we discuss the phenotypic plasticity of macrophages and its functional significance. Finally, we address crosstalk between macrophages and other cell types within the myocardium post-MI, which dictates inflammatory and repair processes. Prospective therapeutic avenues for MI are then suggested.

## Source and distribution of macrophages

### Source of macrophages in the heart

Macrophages, the innate immune cells that make up the mononuclear efferocytosis system, were originally discovered by Elie Metchnikoff in 1882, who described them as capable of engulfing invading pathogens through efferocytosis. They are now considered to mediate various processes, ranging from cytokine production, coordination of efferocytosis, and granulation tissue formation to organ-specific homeostasis [11]. Macrophages are broadly divided into monocyte-derived macrophages and tissue-resident macrophages (RTMs).

#### Monocyte-derived macrophages

Circulating monocytes are produced via hematopoiesis in the bone marrow. Two main monocyte subsets are observed in the blood, classical and non-classical, which were originally found in humans. With the aid of two-color immunofluorescence and flow cytometry, a subset of cells co-expressing CD14 (lipopolysaccharide receptor) and CD16 (FcγRIII) can be observed in human peripheral blood, with a subset of cells expressing Ly-6C later found in mice. Phenotypic analysis showed that these populations correspond in human and mouse to each other, which helped experimentally determine the roles of macrophage populations in human studies [12, 13]. Classical versus non-classical monocytes are distinguished by expression of CD16 (commonly known as CD14^+^CD16^—^) in humans and Ly-6C (Ly-6C^high^) in mice. Meanwhile non-classical monocytes are commonly referred to as CD14 + CD16 + and Ly-6C^low^, respectively [14]. Markers that can distinguish between subsets have been established, including CCR2, MHC-II, HLA-DR, and others (Table 1) [15–25].

**Table 1:** Phenotypic marker of mouse and human monocyte and macrophage subsets as per references [15–25]

Markers	Type	Location	Species	Monocyte	RTM	M1	M2
CD45	Receptor	Cell membranes	Human/Mouse	＋/＋	＋/＋	＋/＋	＋/＋
CD11b	Receptor	Cell membranes	Human/Mouse	＋/＋	＋/＋	＋/＋	＋/＋
CD11c	Receptor	Cell membranes	Human/Mouse	＋/-	-/-	＋/-	-/-
CD68	Receptor	Cell membranes	Human/Mouse	＋/＋	＋/＋	＋/＋	＋/＋
CD80	Receptor	Cell membranes	Human/Mouse	-/-	low/low	＋/＋	＋/＋
CD86	Receptor	Cell membranes	Human/Mouse	＋/＋	-/-	＋/＋	＋/＋
CD206	Receptor、 Lectin	Cell membranes	Human/Mouse	＋/＋	＋/＋	-/-	＋/＋
iNOS	Enzyme	Cytoplasm	Human/Mouse	-/-	-/-	＋/＋	-/-
Arg1	Enzyme	Cytoplasm	Human/Mouse	-/-	＋/＋	-/-	＋/＋
CCR2	Receptor	Cell membranes	Human/Mouse	±/±	-/-	high/＋	low/±
CX3CR1	Receptor	Cell membranes	Human/Mouse	-/±	-/-	low/±	high/＋
IL-6	Cytokine	Secreted	Human/Mouse	-/-	-/-	＋/＋	-/-
IL-10	Cytokine	Secreted	Human/Mouse	-/-	＋/＋	-/-	＋/＋
TNF-α	Cytokine	Secreted	Human/Mouse	-/-	-/-	＋/＋	-/-
CD14	Receptor	Cell membranes	Human	＋	＋	high	＋
CD16	Receptor	Cell membranes、Secreted	Human	±	＋	-	high
HLA-DR	Multi-subunit complex、Receptor	Cell membranes	Human	＋	＋	＋	＋
F4/80	Receptor	Cell membranes	Mouse	＋	＋	＋	＋
Ly-6C	Receptor	Cell membranes	Mouse	high/low	low	high	low
MHC-II	Multi-subunit complex、Receptor	Cell membranes	Mouse	＋	＋	＋	＋
Fizz1	Other	Secreted	Mouse	-	-	-	＋
Ym1	Lectin	Nucleus、Secreted	Mouse	-	-	-	＋

*RTM* tissue-resident macrophage

Classical monocytes are released from the bone marrow and extramedullary hematopoietic sites (such as the spleen), then migrating to the site of injury where they differentiate into macrophages in a CCR2 [chemokine (C–C motif) receptor 2]-dependent manner. Subsequently, these are polarized to a pro- or anti-inflammatory phenotype to regulate tissue inflammation [14, 26]. In contrast, non-classical monocytes are thought to persist in the circulation, and their role in inflammation is less clear, being mainly involved in maintaining homeostasis [11]. For example, non-classical monocytes patrol blood vessel walls and process damaged endothelial cells to preserve blood vessel wall homeostasis in healthy mice [27].

#### RTMs

Most RTMs originate from the yolk sac and fetal liver during embryonic development. Later, they are regulated by M-CSF to mature into RTMs, such as Langerhans cells and microglia [28]. M-CSF renews the RTM pool, maintaining macrophage numbers [29]. RTMs play an important role in shaping and remodeling tissues during development and adulthood. These cells are essential for the maturation of the nervous and vascular systems, contribute to bone and tooth morphogenesis, remove apoptotic cells in the embryo, and coordinate the regeneration of the heart as well as of accessory tissues. In adults, RTMs affect homeostasis and physiological processes, including iron metabolism and transport, hematopoietic regulation, induction of electrical impulses through the heart, tissue remodeling, coronary development, and repair, in addition to providing a protective barrier for joints [30–33]. After tissue injury, the recovery of activated resident macrophages is initially low and is described as a ‘disappearance reaction’. This disappearance can be due to increased tissue adherence, tissue emigration through draining lymphatics, and, possibly, cell death [34]. Upon recovery from the inflammatory episode, RTMs exhibit enhanced proliferation in response to M-CSF in order to repopulate inflamed tissues [35].

The function of RTMs extends beyond immunity. Tissue microenvironments constantly fluctuate in response to external cues, conferring a certain degree of dynamism to RTMs. Therefore, it has been proposed that RTMs should be considered as an integral part of tissues [36]. The factors shaping RTM phenotypes and functions can be organized within four cardinal points: ontogeny, local environment, inflammation status, and time spent in any given tissue [37]. As previously mentioned, RTMs originate from different sources, thus being divided into early yolk sac macrophages, fetal monocytes, or bone marrow-derived monocytes. In addition, RTMs exhibit different phenotypes and functions based on their tissue of residence. Mature peritoneal macrophages were successfully engrafted in the lungs and acquired an alveolar-macrophage-like phenotype [38], indicating that the local environment is a key factor affecting RTM phenotype. Tissues are very dynamic, and the inflammatory environment resulting from mechanical, physical, and biochemical changes is an important aspect affecting RTM phenotype and function. However, current studies cannot adequately recapitulate the complex involvement of RTMs in inflammation. Tissues host a range of RTMs, from monocyte-derived macrophages that are freshly recruited and differentiate within a few hours to those that have resided there for several months, alongside original embryonic-derived macrophages present since birth. Many studies include only one time-point, usually below the age of 10 weeks, so macrophages may not have enough time to fully differentiate and acquire the late programming of RTM populations [37].

Although fate-mapping studies coupled with transcriptomic and epigenetic analyses have greatly improved our understanding of macrophage ontogeny and revealed various differences in gene expression and regulation among different organs, there are still many gaps in our understanding of RTM phenotype and function.

### Distribution of macrophages post-MI

The heart is the vital organ that drives circulation and, thus, the nutrient and oxygen supply throughout the body. As an integral part of the immune system, macrophages play a pivotal role in the initiation, development, and resolution of inflammation following damage to cardiac tissue.

#### Cardiac macrophage populations in healthy individuals

The human heart contains distinct subsets of CCR2^−^ and CCR2^+^ macrophages. The CCR2^−^ subset is predominant in cardiac tissue, while CCR2^+^HLA-DR^neg^ monocytes are relatively rare, found adjacent to blood vessels within areas of dense fibrosis. CCR2^+^ macrophages usually infiltrate areas enriched in type I collagen, such as scars or fibrotic tissue.

CCR2^−^ macrophages represent an RTM population maintained through cell proliferation, whereas CCR2^+^ macrophages are maintained through a combination of monocyte recruitment and cell proliferation. CCR2^−^ and CCR2^+^ macrophages have distinct functions in the heart. CCR2^−^ macrophages are involved in various forms of tissue remodeling, such as coronary development, postnatal coronary growth, and cardiac regeneration. However, the exact functions of CCR2^+^ macrophages within the resting adult heart have not been completely established [39].

#### Changes in cardiac macrophage populations post-MI

Following MI, the macrophage phenotype is subject to change over time, as RTMs are polarized to an anti-inflammatory/M2 phenotype [40]. Moreover, the number of RTMs in the ischemic area drops drastically. Recruited monocytes infiltrate the infarcted myocardium and are transformed into macrophages during the first week after MI [41–43]. Ly-6C^high^ monocytes/macrophages gradually increase, reaching a peak on the third day after MI and accounting for approximately 50% of the total monocyte/macrophage population, in contrast to the low levels of Ly-6C^low^ monocytes/macrophages on the third and fifth days after MI. No increase in the number of these macrophages was observed on day 7 [44]. However, other studies reported that the number of Ly-6C^high^ monocytes/macrophages in the injured myocardium peaks on day 3 (~ 4 × 10^4^ cells/mg tissue) and decreases thereafter (< 0.5 × 10^4^ cells/mg tissue on day 7), whereas the number of Ly-6C^low^ monocytes/macrophages only peaks on day 7 (~ 2 × 10^4^ cells/mg tissue) [10].

#### Changes in macrophages within different cardiac areas post-MI

The post-MI heart can be divided into infarct, peri-infarct, and non-infarct areas. The number of resident macrophages within the infarcted area decreased by approximately 60% (including resident CCR2^−^MHCII^−^TIMD4^+^ and CCR2^−^MHCII^−^TIMD4^−^ macrophages), followed by a slow increase through in situ proliferation [44]. Most of these were replaced by recruited CCR2^+^ Ly-6C^high^ monocytes and CCR2^+^ monocyte-derived macrophages [41].

In the peri-infarct zone, the number of CD68^+^ macrophages increased significantly in the inflammatory phase (24–72 h post-MI), peaked during the repair phase 0–4 days post-MI, and did not decrease significantly in the late stage [45]. CD68^+^LYVE1^–^ macrophages increased on day 2 after MI, whereas the number of CD68^+^LYVE1^+^ macrophages did not change, highlighting the spatial enrichment of resident CD68^+^LYVE1^−^ macrophages in the peri-infarct zone [46]. Although the number of CD68^+^stabilizer-1^+^ macrophages (also known as M2 macrophages) remained unchanged during the inflammatory phase, it increased during the regeneration phase and did not significantly decrease after the 10^th^ day post-MI [45].

In the non-infarct area, there was an increase in the number of CD68^+^stabilin-1^+^ macrophages on days 4–10 after MI [45]. Due to monocyte recruitment and differentiation, the number of macrophages gradually increased at 4 and 8 weeks after MI. Compared with the non-ischemic myocardium, the number of macrophages in the mature infarct scar was lower and decreased with time [47]. To summarize, at the beginning of the inflammatory phase (days 1–3), the number of CD68^+^ macrophages in the infarct area was significantly higher than that in the peri-infarct and non-infarct areas. Subsequently, after days 4–10 of MI, the number of CD68^+^ macrophages in the infarct area decreased but remained higher than that in the peri-infarct and non-infarct areas. During the late phase of MI (after day 10), the number of CD68^+^ macrophages in the peri-infarct area was significantly higher than that in the non-infarcted area [45].

However, most studies examining macrophages after MI neither distinguish between myocardial regions (infarct, peri-infarct, and remote zones) nor specifically investigate the peri-infarct or distal myocardium. Instead, these have focused on the infarct region. Therefore, the recognition that there are site-specific macrophages with differential functions post-AMI may lead to a better understanding of the role of macrophages in MI.

## Phenotype and function of macrophages post-MI

After MI, the left ventricle undergoes wound healing, with overlapping phases of inflammation, proliferation, and repair [48]. Macrophages play an indispensable role in all stages of cardiac repair. During the early inflammatory phase, macrophages exhibit a pro-inflammatory phenotype, producing a variety of pro-inflammatory cytokines, chemokines, and matrix metalloproteinases (MMPs) [49, 50]. During proliferation and repair, macrophages exhibit an anti-inflammatory phenotype and produce a variety of anti-inflammatory, pro-angiogenic, and pro-repair factors [50–52]. Macrophages can also engulf and clear dead cells as well as tissue debris, which is important for the resolution of inflammation.

### Anti-inflammatory and pro-inflammatory phenotypes

Immediately after MI, there is an acute inflammatory phase that involves significant tissue infiltration by neutrophils, monocytes/macrophages, and lymphocytes [53]. The consensus is that macrophage polarization is driven by cues in the tissue microenvironment, which may include cytokines, growth factors, and microorganism-associated molecular patterns. After AMI, circulating monocytes are recruited to the injured heart which differentiate locally into macrophages and differentiated macrophages can be generally subdivided into pro-inflammatory and anti-inflammatory macrophages [10, 54].

Pro-inflammatory macrophages are characterized by efferocytosis, the scavenging of damaged tissue, increased bactericidal activity (including the expression of NOS2), high antigen presention capacity associated with increased MHC-II expression, as well as the release of pro-inflammatory cytokines, such as IL-12 (interleukin-12), IL-23, IL-27, and TNF-α. They also secrete chemokines such as CXCL9 (CXC motif chemokine ligand 9), CXCL10, and CXCL11. Further, pro-inflammatory macrophages express a wide range of MMPs, such as MMP-1, -2, -7, -9, and -12 [11, 55]. Thus, local inflammation is induced to clear dead tissue within the infarcted area. In contrast, anti-inflammatory macrophages produce anti-inflammatory cytokines such as IL-10, chemokines such as CCL17 (C–C motif chemokine ligand 17) [14, 56], and growth factors, including vascular endothelial growth factor and tumor growth factor-beta (TGF-β). Together, these mediators stimulate extracellular matrix production by fibroblasts, cell proliferation, and angiogenesis, thereby promoting tissue remodeling and repair.

In general, the phenotypical transformation of macrophages after infarction is regulated by the local microenvironment. In the early stages of infarction, many pro-inflammatory factors are released to promote macrophage polarization into a pro-inflammatory phenotype. Based on studies involving LPS-induced inflammation, macrophages switch to the M1 phenotype following activation by IFN-β, which requires TRIF-dependent signaling from TLR4 to IRF3 [57]. Interestingly, tenascin-C (TNC) can also promote the polarization of pro-inflammatory macrophages through TLR4 and weaken the effect of IL-4, upregulating the mRNA expression of interferon regulatory factor 4 and thereby inhibiting anti-inflammatory polarization [58].

In addition to the classical LPS-induced inflammatory phenotype, several cytokines have been recently shown to regulate macrophage polarization toward a pro-inflammatory phenotype, with multiple signaling pathways implicated. For example, Lgr4 (a member of the leucine-rich repeat-containing G protein-coupled receptor family) promotes AP-1 activity by enhancing CREB-mediated c-Fos, Fosl1, and Fosb transactivation, leading to intrinsic pro-inflammatory activation of macrophages within the infarct region [59]. Additionally, MST1/2 (mammalian STE20-like protein kinase 1/2) is a major component of the Hippo signaling pathway and regulates the macrophage-based immune response to bacterial infection. MST1 defects promote pro-inflammatory macrophage polarization via the MST1–5-LOX–LTB4–BLT1 axis and hinder cardiac repair after MI [60].

Over time, the infarct area gradually shifts to the repair stage. Macrophages are polarized into the anti-inflammatory phenotype to promote tissue remodeling and repair. The roles of IL-4- and IL-13-mediated signal transduction in anti-inflammatory macrophage polarization have been confirmed. They act through the IL4Rα-JAK signal transducer and activator of transcription 6 (STAT6) pathway, which regulates many pro-inflammatory macrophage-related genes in mice, including arginase 1 (Arg1), macrophage mannose receptor 1 (Mrc1, also known as CD206), resistin-like-α (Retnla, also known as Fizz1), and chitinase 3-like 3 (Chi3l3, also known as Ym1) [61]. Crosstalk between the peroxisome proliferator-activated receptors (PPAR)-γ and the IL4–STAT6 axis may collectively regulate the M2 phenotype. A second crosstalk pathway has been described, involving IL-4-mediated stimulation of PPAR-γ activation through the synthesis of putative endogenous PPAR-γ ligands [62]. Additionally, Smad3 is implicated in the anti-inflammatory transition of infarct macrophages, mediating the phagocytosis-associated induction of PPARs [63]. Interestingly, a recent study showed that IL-6 promotes the expression of Hamp, which encodes hepcidin, through STAT3 activation. Hepcidin is an upstream repressor of IL-4 and IL-13. Importantly, the absence of hepcidin in macrophages promotes IL-6 to activate IL-4 and IL-13 secretion through phospho-STAT3, which is a prerequisite for cardiac regeneration [64].

In addition to the classical IL-4/IL-13, various factors that can modulate macrophage polarization have been identified. These include MMP, C/EBP*β*, myonectin, Smad3, and S100A9. Under MMP-28 knockout, IL-4 does not significantly stimulate macrophage polarization toward an anti-inflammatory phenotype [65]. In mouse cardiomyocytes (CMs) overexpressing Nox4, an increase in cardiac macrophages was noted, in addition to an evident change toward the anti-inflammatory phenotype (particularly in the non-infarcted region) after ischemia/reperfusion. Interestingly, the effect of Nox4 may be related, at least in part, to changes in MMP-2 activity during MI repair [66].

It is important to note that cAMP-responsive element-binding protein (CREB) may act as a pivotal transcription factor in macrophage polarization by promoting anti-inflammatory-associated genes while repressing pro-inflammatory activation. A study showed that C/EBP*β* specifically regulates anti-inflammatory genes (including Arg1, IL10, and Mrc1) when its expression is induced by another bZIP family transcription factor, CREB [67]. This response was similar to LPS stimulation, which is mediated by the p38 mitogen-activated protein kinase (p38, also known as MAPK14), mitogen- and stress-activated kinase 1 (MSK1, also known as RPS6KA5), and MSK2 (also known as RPS6KA4). CREB induced the expression of IL-10 and dual-specificity protein phosphatase 1 (DUSP1), while inhibiting the expression of pro-inflammatory genes associated with M1 macrophage activation [68, 69]. Similarly, myonectin is a myokine that is upregulated in skeletal muscle and blood by exercise. It suppresses the inflammatory response to LPS in cultured macrophages through the S1P/cAMP/Akt-dependent signaling pathway [70].

S100A9 was originally defined as a pro-inflammatory alarmin. Its short-term inhibition during the inflammatory phase can suppress systemic and myocardial inflammation, thus improving cardiac function after MI [71]. A recent study found that S100A9 can activate the transcription factor Nur77 (Nr4a1, nuclear receptor subfamily 4 group A member 1) and promote the differentiation of inflammatory Ly-6C^high^ monocytes into reparative Ly-6C^low^MerTKhi macrophages [72].

After polarization, macrophages secrete not only cytokines but also exosomes with distinct functions. As natural nanoparticles, exosomes have shown high biocompatibility and specific targeting ability, thus being widely employed as imaging agents and drug carriers in recent years [73]. For example, M1 macrophage-derived exosomes were used as nanoparticle coatings to target drug-resistant and metastatic tumors [74]. Unfortunately, there are no studies on exosome-encapsulated drugs to treat MI, necessitating research into the matter. For example, exosomes containing vascular growth factors can promote post-infarction angiogenesis, thereby reducing the infarct area. Alternatively, exosomes may be used to deliver TGF-β for the promotion of fibrosis and prevention of heart rupture after MI.

### Efferocytosis by macrophages

Macrophages phagocytose dead cells and debris through a process called efferocytosis (removal of apoptotic cells) [53]. It is a prerequisite for the resolution of inflammation. Efferocytosis can be divided into four main steps [75]. In the first step, apoptotic cells release ‘find-me’ signals to attract phagocytes [76]. These include nucleotides adenosine triphosphate and uridine triphosphate, which are sensed by the macrophage purinergic receptor P2Y2 (22), or the lipids lysophosphatidylcholine (23) and sphingosine1-phosphate, which bind to the macrophage G-protein-coupled receptors G2A and S1P1-5, respectively [77–79]. In addition, the recognition of dead cells by macrophages may be promoted by the interaction between intercellular adhesion molecule 3 (ICAM3 or CD50) on dead cells and CD14 on macrophages as well as the interaction between thrombospondin (TSP1) and CD36 [80, 81].

The second step is the recognition of ‘eat-me’ signals on the surface of apoptotic cells, which promotes specific recognition by phagocytes and subsequent internalization [82]. Phosphatidylserine (PS) is the most common ‘eat-me’ signal. During apoptosis, this phospholipid is presented outside the cell membrane and can be directly recognized by specific efferocytosis receptors on macrophages, such as MerTK (myeloid-epithelial-reproductive tyrosine kinase), CD36, integrins αvβ3 and αvβ5, TIM-1,4 [T-cell membrane protein (Tim) family], BAI1 (brain angiogenesis inhibitor 1), and stabilin-2. PS can also be recognized indirectly by receptor tyrosine kinases Tyro-3, Axl, and Mer that bind to efferocytosis receptors through bridging molecules [for example, MFGE8 (milk fat globule-EGF factor 8 protein), developmental endothelial locus-1 (DEL-1), galectin-3 (Gal-3), growth arrest-specific factor 6 (Gas6), and protein S)] [83–85].

In the third step, after the soluble ligand binds to the receptor, the membrane invaginates, and vesicles containing the receptor and the ligand/cargo from the plasma membrane lyse [86]. Time delay studies show that vesicles move through a series of stages (coated with GTPases Rab5 and Rab7) in cells, leading to progressive acidification, with the cargo being separated from the receptor, which can then be recycled back to the membrane, while the contents can be transported to lysosomes in cells for degradation [87].

The fourth step, considered a consequence of efferocytosis, involves the release of anti-inflammatory cytokines (TGF-β, IL-10, and IL-12) or inflammatory cytokines (IL-1β, TNF, and IL-6) secreted by phagocytes that are triggered during recognition and processing [88–90]. However, the specific mechanisms involved are unclear.

In addition, it has been found that RTMs effectively uptake vesicles produced by injured myocardial cells through the receptor MerTk, thus maintaining cardiac homeostasis [91]. The efferocytosis capacity of RTMs is decreased with legumain (Lgmn) deficiency. This may be related to the function of Lgmn in mediating the conjugation of LC3-II and phagosomes, resulting in phagosome-lysosome fusion and CM degradation, PARP2 cleavage, and removal of its extracellular FLAG epitope to increase cytosolic calcium [92].

## Crosstalk between macrophages and cardiac cells post-MI

The heart is composed of various cell types, such as myocardial cells, fibroblasts, immune cells, and vascular endothelial cells [93]. Each cell type executes a specialized function, while also participating in crosstalk with other cell types in order to maintain cardiac homeostasis. This crosstalk has a considerable influence on MI pathogenesis and prognosis. Hereafter, we elaborate on the possible mechanisms underlying crosstalk between macrophages and cardiac cells.

### Crosstalk between macrophages and CMs

CMs are the basic functional cells of the heart. Upon MI, many CMs die, forming the infarct area. In the subsequent wound-healing process, dead CMs must be effectively cleared to prevent secondary necrosis and long-term inflammation. This clearance is particularly important in the heart as the ineffective clearance of dead CMs may lead to loss of collateral muscle cells and infarct expansion [94, 95]. Therefore, efferocytosis by macrophages is instrumental for the clearance of dead and apoptotic CMs. The recognition of dead cells during efferocytosis is a multi-step process that requires the chemotactic recruitment of macrophages, receptor-mediated binding of target cells and macrophages at efferocytosis synapses, as well as the internalization and breakdown of dead cells [96, 97].

Many studies have shown that specific efferocytosis receptors on macrophage membranes are key to regulating CM efferocytosis. After MI, macrophages have been shown to identify CMs via the MerTk receptor [98–100]. During homeostasis, CMs release subcellular particles containing defective mitochondria, called exophers. These are captured and cleared by surrounding RTMs via the MerTk receptor, in a process that prevents inflammasome activation and blocks autophagy, constituting an important mechanism for maintaining cardiac homeostasis. When macrophages or MerTk are ablated, cardiac metabolic function is impaired [91]. Shuang et al. showed that CMs can induce shedding of the apoptosis receptor MerTk, leading to reduced efferocytosis after MI, yet the mechanism is unclear [97].

In addition to the classical apoptotic receptor MerTk, the integrin-related proteins CD47 and CD72 also affect the crosstalk between macrophages and CMs after MI. CD47 expression in CMs was shown to increase after MI, impairing efferocytosis by macrophages via the CD47-SIRPα axis. Further, SIRPα enhanced the efferocytosis of dying CMs when CD47 antibodies or agonists were used to block CD47 on CMs or engage CD47 on macrophage membranes, respectively [95].

Interestingly, after MI, macrophages not only perform efferocytosis to ingest apoptotic or damaged CMs, but also promote CM proliferation and regeneration. The hearts of newborn mice can completely regenerate after MI through orchestrated waves of inflammation, matrix deposition, and remodeling that involve CM proliferation. The downstream signaling pathways involved include the Hippo-Yap, Jak1-STAT3, MekErk1/2, and Notch [101–103]. With regard to upstream signals, Yan Dong Li et al. found that oncostatin M (OSM), a cytokine secreted by macrophages, is a key regulator of CM proliferation and cardiac regeneration, acting through the gp130/Src/Yap-Notch and Yap-ctgf/Areg pathways, which are independent of Hippo signaling [104].

In addition, CCR2^−^ macrophages can interact with CMs through focal adhesion complex markers (such as β-integrins) and can experience an increase in LV pressure as well as a subsequent increase in myocardial wall stress. TRPV4 (transient receptor potential vanilloid 4) is activated by such mechanical stimulation to promote the expression of macrophage growth factors, which is independent of MYD88 and TRIF [30].

**Fig. 1:**
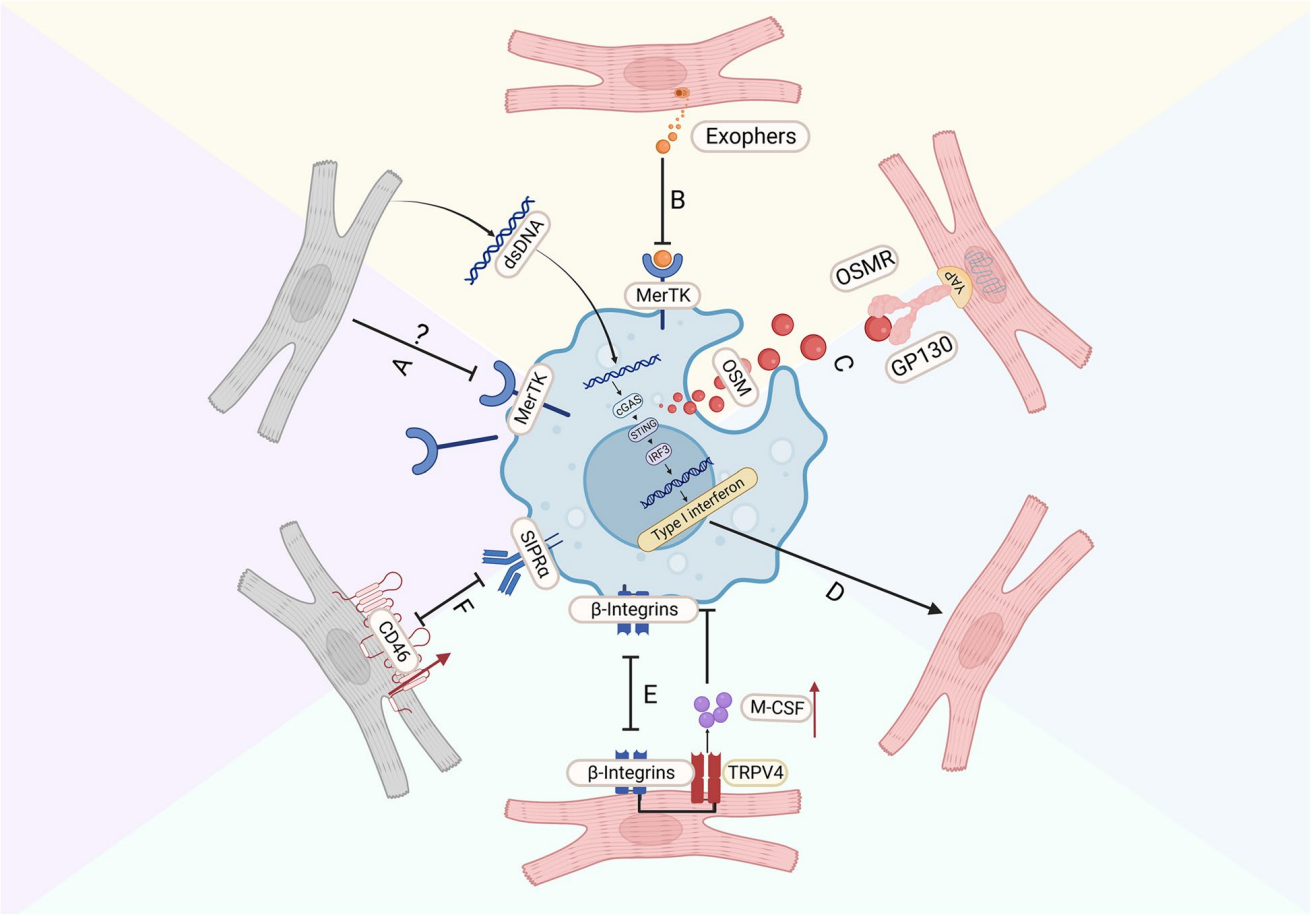
Crosstalk between macrophages and cardiomyocytes (CMs). **A** Dead CMs induce shedding of the MERTK receptor. **B** Macrophages phagocytose exophers to maintain homeostasis. **C** Macrophages secrete OSM to promote CM proliferation through gp130/Src/Yap-Notch and Yap-ctgf/Areg pathways. **D** The dsDNA from CMs that have undergone cell death stimulates the cGAS-STING-IRF3 pathway to induce the apoptosis of healthy CMs. **E** Macrophages interact with CMs via focal adhesion complex markers (such as β-integrin) and activate TRPV4 to promote the expression of M-CSF by sensing left ventricular pressure.** F** CD47 expression on CMs impairs the efferocytosis by macrophages

It is worth noting that during MI, dsDNA from CMs that have undergone cell death stimulates the cGAS-STING-IRF3 pathway in bone marrow-derived macrophages (BMDMs) to then potentially induce the apoptosis of healthy CMs. When H-151 is used to inhibit this pathway, macrophage-mediated CM apoptosis is reduced. H-151 inhibits the type I interferon response in BMDMs via cGAS-STING-IRF3 [105]. In addition, a study showed that IL-7 could not directly induce cell apoptosis after ischemia–reperfusion but could induce CM apoptosis through macrophages and enhance their migration in vitro. However, its role in MI has not yet been investigated [106] (Fig. 1).

### Crosstalk between macrophages and fibroblasts

The heart acts as an effective muscle pump, essentially a syncytium of CMs connected to a network of structural proteins consisting mainly of type I collagen fibers with the tensile strength of steel. The stability of this extracellular matrix and its dynamic balance with CMs arise through the gradual transformation (i.e., synthesis and degradation) of interstitial fibroblasts and their collagen [93]. In adult mammals, the sudden massive loss of CMs after MI exceeds their limited regenerative capacity [107], and the structural integrity of the myocardium is maintained by activated fibroblast-like cells, with these phenotypically transformed, α-smooth muscle actin microfilament-expressing cells referred to as ‘myofibroblasts’. In the later stages of cardiac regeneration, myofibroblasts induce a healing response, including the formation of collagen fibers that eventually form scar tissue [108]. Although initial reparative fibrosis is essential for preventing ventricular wall rupture, excessive fibrosis in the infarcted and peri-infarcted areas leads to oversized scar tissue that can progressively impair heart function and eventually result in heart failure [109, 110]. Therefore, it is important to regulate fibrosis in order to improve the prognosis of MI. During the wound-healing stage, recruited macrophages regulate fibrosis within the injured area. These express renin and angiotensinogen-converting enzyme (ACE), producing angiotensin II through autocrine action at the injury site, thereby upregulating and activating TGF-β_1_, which triggers the appearance of myofibroblasts at the injury site. Angiotensinogen II produced by macrophages also binds to AT1 receptors on myofibroblasts and upregulates TGF-β_1_ to regulate the deposition of matrix proteins and inhibit matrix degradation in order to promote tissue repair and scar formation [108].

In addition to the classical renin–angiotensin–aldosterone system and TGF-β_1_, macrophage subsets can produce large amounts of pro-fibrotic growth factors, such as IL-10, IGF-1, platelet-derived growth factors, and fibroblast growth factors [111–113]. A recent study found that lgr4 is a leucine-rich repeat-containing G protein-coupled receptor. It promotes AP-1 activation in inflammatory macrophages by enhancing CREB-mediated *c-Fos*, *Fosl1*, and *Fosb* transactivation [59]. Surprisingly, macrophages may be transformed into fibroblast-like cells after MI, yet the specific mechanism of how this occurs has not been determined [114].

An increasing number of studies have highlighted the role of macrophage-derived exosomes in fibrosis. M2-like macrophages activate the circUbe3a/miR-138-5p/Rhoc signaling axis, interacting with fibroblasts via the release of circUbe3a-enriched endocytic membrane-derived vesicles that mediate intracellular communication through the delivery of proteins and RNA. This may also affect cardiac fibrosis after MI [115]. As previously described, fibrosis inhibition is essential for improving cardiac function after MI. Abe et al. found that after MI, Ly-6C^high^ macrophages secrete HIF-1 to target the *OSM* gene, thereby inhibiting the ERK1/2-SMAD2-TGFβ_1_ axis-mediated cardiac fibroblast activation [116]. Alternatively, macrophages secrete mir-155-enriched exosomes that are taken up by cardiac fibroblasts, inhibiting their proliferation through the downregulation of Sos1 expression [117].

**Fig. 2:**
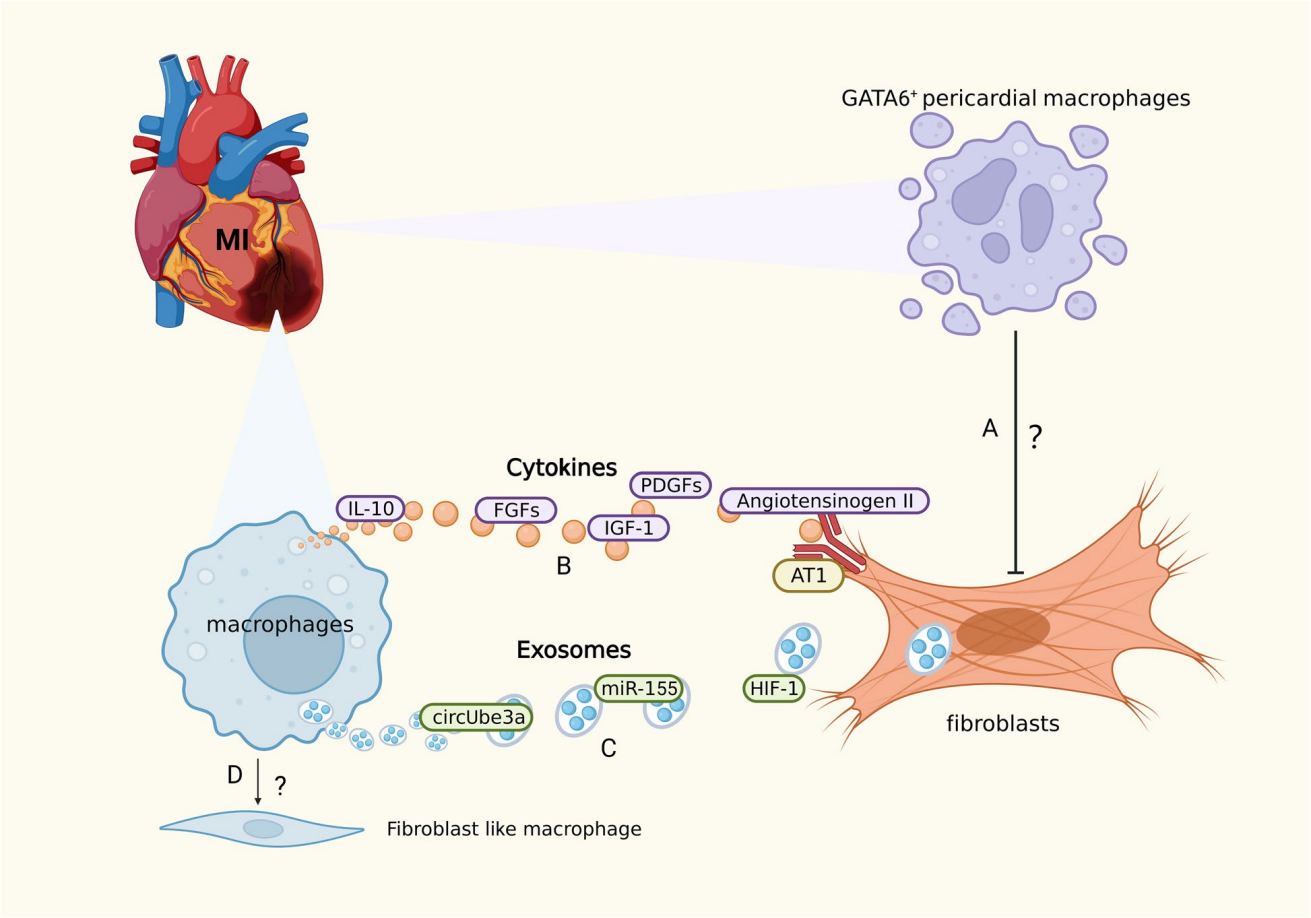
Crosstalk between macrophages and fibroblasts. **A** The role of Gata6^+^ pericardial macrophages (GPCMs) remains controversial. **B** Macrophages secrete cytokines (such as IL-10, IGF-1, FGFs, PDGFs, angiotensinogen II) to promote fibrosis. **C** Macrophages secrete exosomes enriched with circUbe3a, HIF-1, or mir-155 to inhibit fibrosis. **D** Macrophages may be transformed into fibroblast-like cells

Interestingly, in addition to RTMs and monocyte-derived macrophages recruited after infarction, Gata6^+^ pericardial macrophages (GPCMs) are also involved in cardiac fibrosis after MI, yet their role remains controversial. Deniset et al. found that GPCMs were gradually recruited to the infarcted area after MI and altered their phenotypes. Adverse fibrosis in the infarcted region was increased in Gata6-knockout mice. It is therefore speculated that GPCMs can prevent fibrosis in MI to a certain extent [118]. Jin et al. employed genetic lineage tracing to re-assess GPCMs during cardiac injury and repair. They developed a dual recombinase-mediated genetic tracing system to specifically label GPCMs, demonstrating GPCM accumulation on the infarct surface, without penetration into the myocardium. There were no significant changes in myocardial fibrosis or cardiac function following ablation of GPCMs or knockout of Gata6, in the context of MI. Therefore, the authors suggest that GPCMs do not prevent cardiac fibrosis nor contribute to repair [119]. The widely divergent views of GPCMs proposed by the two groups may stem from different experimental techniques and insufficient sample sizes. Further studies are therefore needed to determine the specific role of GPCMs after MI (Fig. 2).

### Crosstalk between macrophages and other immune cells

Accumulating evidence shows that a series of finely regulated inflammatory reactions occur after MI [10]. While the inflammatory response was initially thought to be detrimental, more studies have found that the immune system drives repair and tissue remodeling after AMI, determining the degree of myocardial injury and the subsequent disease course [120, 121]. After MI, damaged CMs release damage-related molecular patterns (DAMPs), cytokines, and chemokines, resulting in the recruitment of a large number of immune cells to the myocardium [122].

Neutrophils first reach the damaged cardiac tissues after MI, peaking in number on the first day after ischemia [123]. Although neutrophils initially contribute to the clearance of cell debris, they secrete inflammatory mediators that lead to tissue damage and further immune cell aggregation [124]. Ly-6C^high^ monocytes/macrophages in the spleen are recruited and activated by neutrophils in an angiotensin II-dependent manner. When neutrophils are depleted, they can reduce the recruitment of Ly-6C^high^ monocytes/macrophages from the spleen, and cardiac macrophages mostly proliferate in a repair phenotype, inducing significant fibrosis [125]. Moreover, neutrophil gelatinase-associated lipocalin (NGAL) in the neutrophil secretome influences efferocytosis by affecting the expression of efferocytosis receptor MertK on the membrane of cardiac macrophages. In the absence of NGAL, MerTk expression on the cardiac macrophage membrane is reduced, which impairs efferocytosis [125]. Except for NGAL, the DNA of neutrophil extracellular traps primes Mertk^−^MHC-II^lo−int^ macrophage polarization via the TRL9 pathway, yet the specific mechanism remains unclear [122]. Conversely, Ly-6C^low^ macrophages produce MMP-12 during the repair stage, reducing the levels of neutrophil-attracting chemokines (including CXCL1, CXCL2, and CXCL5) to limit neutrophil infiltration in infarcted hearts and promote wound healing [126]. Moreover, IL-4 may be a key factor in the crosstalk between macrophages and neutrophils. Daseke et al. demonstrated that exogenous IL-4 downregulated neutrophil pro-inflammatory markers (e.g., CCL3, IL12A, and TNF-α) to stimulate the anti-inflammatory response in macrophages (upregulating Arg1 and Ym1) and mediate apoptotic neutrophil clearance. Treatment with IL-4 enhanced the efferocytosis of neutrophils by macrophages within the infarcted area, thereby alleviating inflammation [127].

T lymphocyte populations are broadly subdivided into helper CD4^+^ T lymphocytes and cytotoxic CD8^+^ T lymphocytes [128]. The former participate in MI through crosstalk with macrophages. In addition to the Th1 (secrete INF-γ and TNF) or Th2 (secrete IL-4 and IL-13) phenotypes, post-MI CD4^+^ T cells can also be classified into ‘effector’ (Teff; Foxp3^−^) and ‘regulatory’ (Treg; Foxp3^+^) subsets based on Foxp3 expression. Th1 cells promote pro-inflammatory macrophage polarization, while Th2 and Treg cells (secrete IL-10 and TGF-β) promote anti-inflammatory macrophage phenotypes, thereby affecting healing and scar formation [129]. Jia et al. found that, after MI, Treg cells secrete more IL-35 than under normal conditions, with IL-35 stimulating transcription of _CX3_CR1 (C-X3-C motif chemokine receptor 1) and TGF-β_1_ in macrophages through GP130, IL12Rβ2, as well as phosphorylation of STAT1 and STAT4, which subsequently promotes Ly-6C^low^ macrophage survival and extracellular matrix deposition. This enhances fibrosis after MI and reduces the left ventricular rupture rate [130].

In addition to classical neutrophils and T lymphocytes, eosinophils and basophils have also been observed following MI. Eosinophils are another subgroup of granulocytes whose role in MI is usually ignored. Eosinophil recruitment occurs on day 4 after MI [131]. It may be related to the transition from MI to cardiac repair. When eosinophils are deficient, the availability of IL-4, IL-5, IL-13, and IL-10 in the infarct area is significantly reduced, whereas the expression of the pro-inflammatory mediators IL-18, CCL5, and TNF-α is increased, and macrophage polarization into an anti-inflammatory phenotype is impaired [132]. Apart from this, Xu et al. found that macrophage-secreted IL-5 after MI increased the aggregation of eosinophils, which in turn induced the transition of BMDMs toward a CD206^+^ phenotype via the IL-4/STAT6 axis, promoting the recovery of cardiac dysfunction [133].

**Fig. 3:**
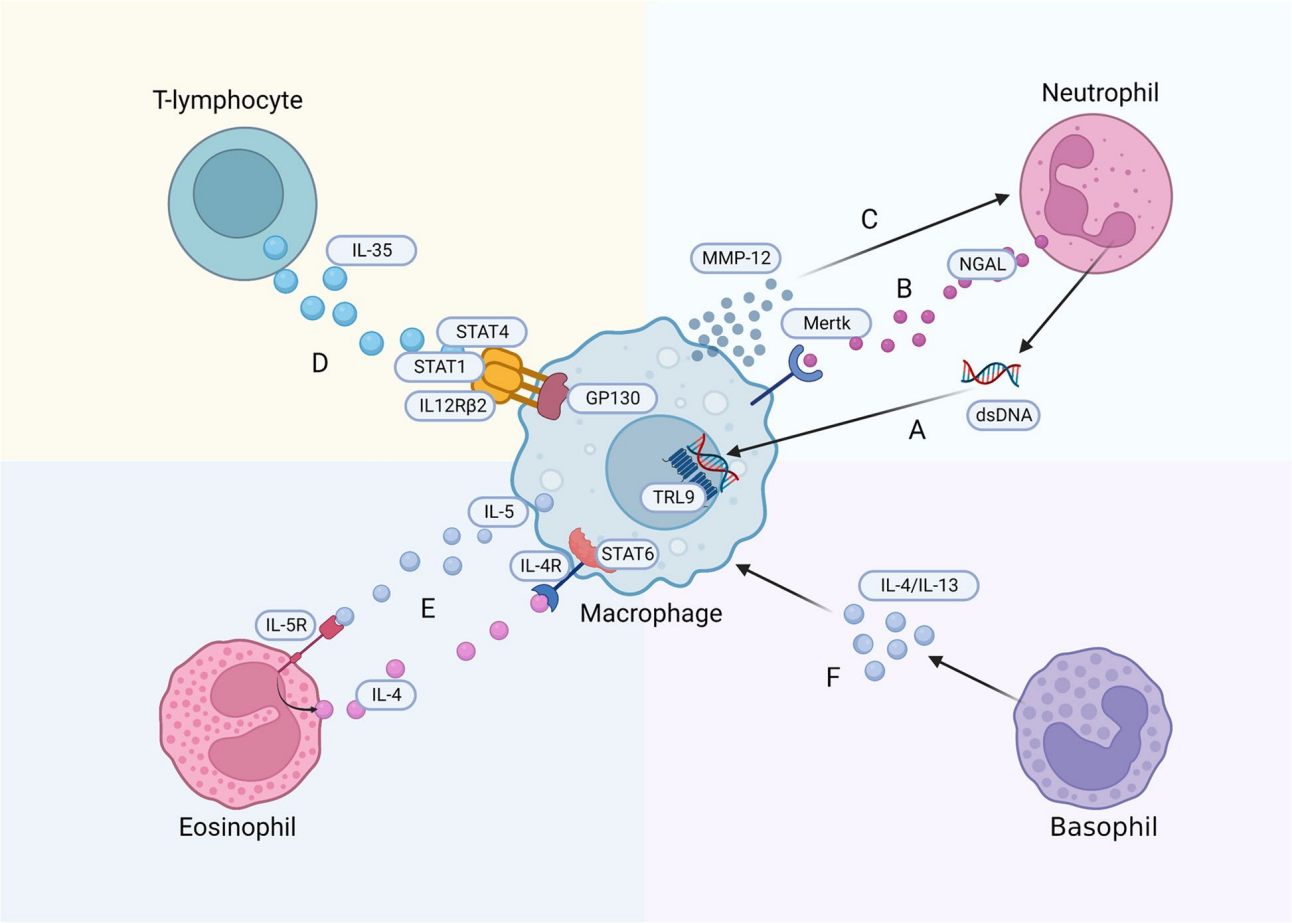
Crosstalk between macrophages and immune cells. **A** The neutrophil DNA primes macrophage polarization via the TRL9 pathway. **B** Neutrophils affect efferocytosis by secreting NGAL which affects expression of the MertK receptor. **C** Macrophages produce MMP-12 to reduce neutrophil infiltration. **D** T cells secrete IL-35, which upregulated the expression of CX3CR1 and TGF-β1 in macrophages by inducing GP130 signaling via IL12Rβ2 and phosphorylation of STAT1 and STAT4. **E** IL-5 secreted by macrophages increases the aggregation of eosinophils, which induces phenotypic transformation of macrophages through the IL-4/STAT6 axis. **F** Basophils secrete IL-4/IL-13 to regulate phenotype transformation of macrophages

The number of basophil granulocytes infiltrating the infarcted hearts of mice peaked between days 3 and 7 after MI. Antibody-mediated and genetic depletion of basophils compromises cardiac function and enhances scar thinning after MI [134]. This may be related to the phenotypic transformation of macrophages, which is regulated by basophils. The expression of macrophage phenotypic markers (such as IL-12β, IFN-γ, and arginase-1) and polarization is modulated via production of IL-4/IL-13 cytokines within the damaged heart after MI [134–136] (Fig. 3).

### Crosstalk between macrophages and vascular endothelial cells

After MI, many myocardial cells are lost due to ischemia. Immediate restoration of the blood supply (such as percutaneous coronary intervention) is the main method of treating MI and can maximize the survival rate of patients by preserving cardiac function. However, there are many problems with reperfusion treatment in clinical practice, including ischemia–reperfusion injury and missing the optimal timing for treatment. Therapeutic angiogenesis significantly promotes repair of the myocardial infarct and prevents adverse ventricular remodeling. The crosstalk between macrophages and cardiac endothelial cells plays a crucial regulatory role in vascular remodeling after MI [137–139].

The classical mechanism underlying macrophage-mediated angiogenesis after MI is through regulation of vascular endothelial growth factor levels. The expression of annexin A1 (ANXA1) in neutrophils infiltrating the damaged tissue is increased, stimulating the polarization of macrophages toward a repair phenotype and releasing large amounts of VEGF-A [140]. Angiogenesis may be later induced through multiple pathways, such as ROS/ER, ROS/START3, and VEGFR-2/p38MAPK signaling [141–143].

With respect to coronary artery development, CCR2^−^ macrophages are recruited into the coronary artery vessels at the beginning of perfusion to mediate coronary artery remodeling by selectively expanding perfusion vessels. This may be related to the pro-angiogenic properties of embryonic-derived macrophages mediated by the insulin-like growth factor (IGF) [33]. Moreover, Wong et al. demonstrated that RTMs physically interact with adjacent CMs in adult mouse hearts, with mechanical sensing through a TRPV4-dependent pathway. This regulates IGF1 expression in CCR2^−^ cardiac macrophages and the formation of coronary arteries in dilated cardiomyopathy, yet its role in MI remains elusive [30].

MMPs are a family of zinc-dependent endopeptidases that have traditionally been associated with the degradation and turnover of ECM (extracellular matrix) components. It is now known that MMPs, directly and indirectly, regulate cell behavior and the microenvironment through the proteolytic processing of various factors, including membrane receptors and growth factors [144]. Membrane type 1 matrix metalloproteinase (MT1-MMP/MMP14) is the first membrane-anchored MMP to be described, involved in the degradation of a spectrum of structural matrix proteins (including collagens I, II, III, fibronectin, and laminin), the proteolytic processing of growth factors and cytokines (e.g., TGF-β and SDF-1), as well as the activation of other MMPs (e.g., MMP2) [145]. Recent studies show that myocardial macrophages increase the expression of MMP14 (MT1-MMP) after MI, activating TGF-β_1_ and leading to paracrine SMAD2-mediated signaling in endothelial cells as well as the endothelial-mesenchymal transition (ENDMT). Macrophage-specific targeting of MT1-MMP attenuates post-MI cardiac dysfunction, reduces fibrosis, and preserves the cardiac capillary network [146].

Macrophages not only promote, but also inhibit endothelial cell proliferation. Liu et al. demonstrated that pro-inflammatory M1-like macrophages release exosomes (M1-Exos) rich in miR-155 following MI. MiR-155 downregulates target genes by reaching endothelial cells, including Rac family small GTPase 1 (RAC1), p21 activated kinase 2 (*PAK2*), Sirtuin 1 (*Sirt1*), and protein kinase AMP-activated catalytic subunit alpha 2 (AMPKα2), to inhibit Sirt1/AMPKα2–endothelial nitric oxide synthase. Furthermore, RAC1–PAK2 signaling suppresses the angiogenic capacity of endothelial cells, aggravates myocardial injury, and inhibits cardiac regeneration [137].

Endothelial cells also exert certain effects on macrophages. Recent evidence has shown that after MI, the increase in Angpt2 expression in endothelial cells is directly regulated by FOXO1, which antagonizes Tie2, thus suppressing PI3K/Akt signaling while enhancing NF-κBp65 and FOXO1 expression. Therefore, increased FOXO1 transcriptional activity forms a positive feedback loop with Angpt2 and has a sustained effect on endothelial cells. In macrophages, the Angpt2/ integrin α5β1/ERK– signaling pathway plays an important role in the pro-inflammatory polarization of macrophages through autocrine and paracrine pathways [147]. Surprisingly, endothelial cells connect with macrophages through the highly expressed sphingosine 1-phosphate receptor 1 (S1pr1) in a contact-dependent manner, promoting the proliferation of anti-inflammatory macrophages in damaged cardiac tissue through the S1P/S1PR1/ERK/CSF1 pathway, thereby improving adverse cardiac remodeling after MI [148].

**Fig. 4:**
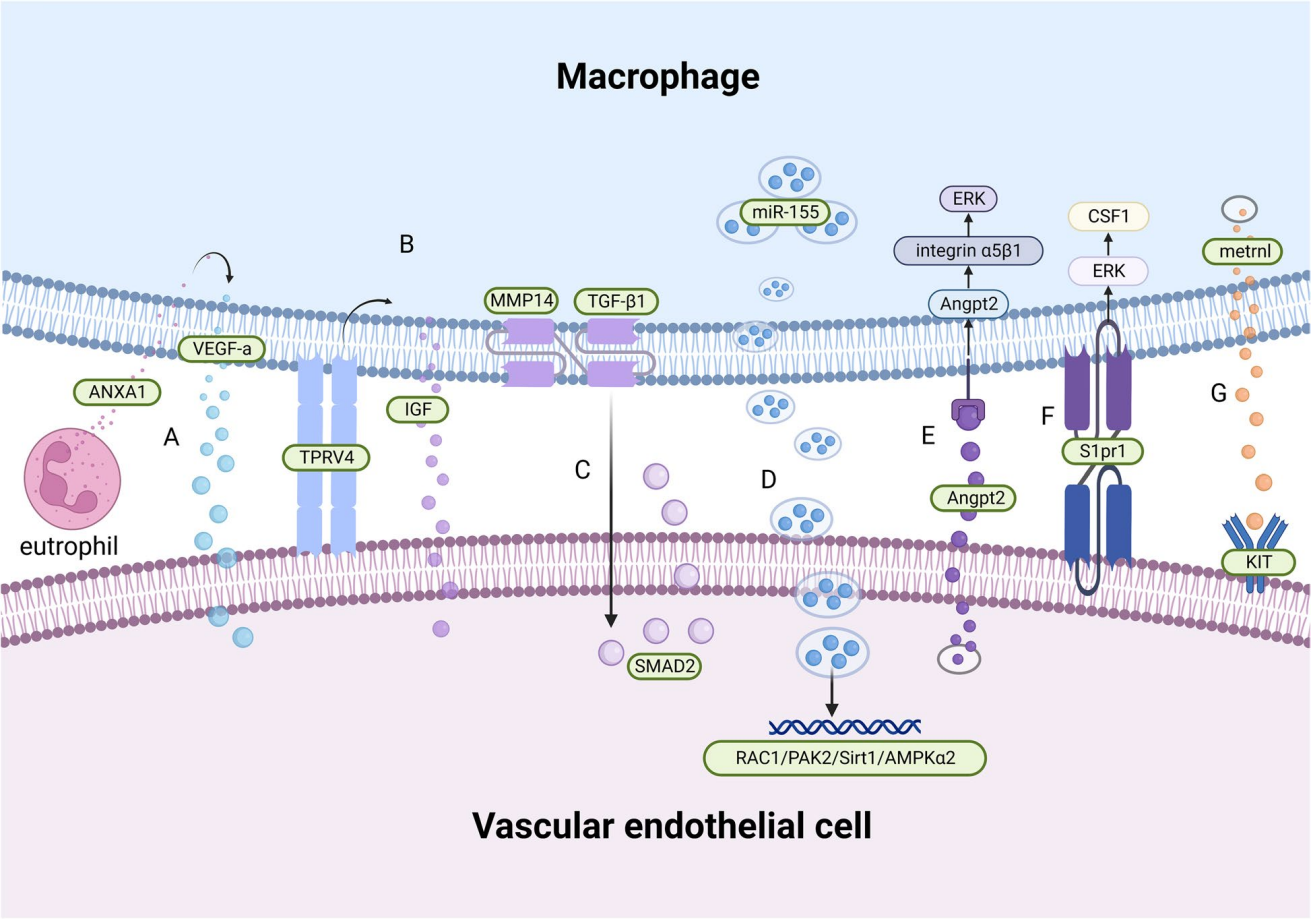
Crosstalk between macrophages and vascular endothelial cells. **A** Neutrophils secrete ANXA1, which stimulates VEGF-A secretion by macrophages, promoting angiogenesis. **B** Macrophages physically interact with CMs through a TRPV4-dependent pathway to regulate the expression of IGF1. **C** Macrophage MMP14 expression induced the activation of TGF-β1 to stimulate SMAD2 activity in a paracrine manner. **D** MiR-155-enriched exosomes secreted by macrophages downregulate endothelial target genes, including RAC1, PAK2, Sirt1, and AMPKα2. **E** Endothelial cells secrete Angpt2 to regulate macrophages through the Angpt2/integrin α5β1/ERK signaling pathway. **F** Endothelial cells interact with macrophages in a contact-dependent manner through the highly expressed sphingosine 1-phosphate receptor 1 (S1pr1) to promote the proliferation of anti-inflammatory macrophages via the S1P/S1PR1/ERK/CSF1 pathway. **G** Metrnl (meteorin-like) binds the stem cell factor receptor KIT on endothelial cells to mediate angiogenesis

In addition, Reboll et al. identified a new macrophage-derived cytokine, metrnl (meteorin-like). It is the driving factor of post-infarction angiogenesis and mediates its effects via binding to stem cell factor receptor KIT (KIT receptor tyrosine kinase) on the surface of endothelial cells [149] (Fig. 4).

### Crosstalk between macrophages and lymphangiogenesis

The lymphatic vasculature is a blind-ended network crucial for tissue-fluid homeostasis, immune surveillance, and lipid absorption from the gut. Recent research has shown that the cardiac lymphatic system is associated with the remission of inflammation after MI [150]. In MI, the expression of the pre-lymphangiogenic factor vascular endothelial growth factor C (VEGF-C) is induced, which triggers cardiac lymphangiogenesis, thereby improving cardiac function [151]. Interestingly, macrophages, especially CD11b^+^ macrophages, secrete VEGF-C and promote lymphangiogenesis during inflammation [152]. However, studies on the crosstalk between macrophages and the lymphatic system have been limited. Glinton et al. reported that cardiac macrophages promote healing by enhancing myocardial lymphangiogenesis. With regard to the mechanism, CD36-dependent exocytosis activates STAT6, leading to the production of VEGF-C by macrophages and promoting myocardial lymphangiogenesis following MI [153]. The role of lymphangiogenesis after MI cannot therefore be neglected, with this process representing a potential therapeutic target.

## Conclusions

Since their initial discovery by Elie Metchnikoff, macrophages have long been generally considered immune cells that primarily phagocytosis. Roles for macrophages in the maintenance of cardiac homeostasis are now emerging. In particular, the clearance of dead CMs in the acute phase as well as their proliferation and regeneration during the tissue repair process involve crosstalk that regulates inflammation, fibrosis, angiogenesis and lymphangiogenesis. In addition, increasing attention has been paid to the specific role of macrophage-secreted exosomes. The various signaling pathways involved in the above-described intracellular crosstalk are yet to be comprehensively explored, as are various other aspects, such as the influence of age and comorbidities have not been fully reflected, which are not reflected in mouse studies.

Furthermore, as macrophage play key roles in the inflammation, proliferation, and tissue repair after MI, they represent a potential therapeutic target. For example, macrophage-targeted gene therapy has great prospects in remodeling the microenvironment of the injured site and reversing damage to the inflammatory site. Further, dually transfecting polyplexes of macrophages and tumor cells have been proposed for cancer treatment [154]. However, macrophages are difficult to transfect, and non-specific delivery would inevitably cause unwanted systemic side effects [155]. The use of macrophage-derived exosomes as drug carriers, the design of biomimetic nanoparticles with macrophage membrane protein engineering, and the generation of nanoparticles wrapped with a macrophage membrane represent ways for achieving targeted delivery [74, 156]. It should be noted that the structural design of such nanotherapeutics is often very complex, which complicates reproducibility and safe preparation. PROTAC may be employed for target protein degradation as it has been previously used to inhibit the polarization of M2 macrophages for the treatment of glioma [157]. However, it is seldom studied in the field of cardiovascular research. Taken together, advances in gene therapy, exosome, and PROTAC technology may enable their wide applicability clinical practice. However, we still lack a comprehensive understanding on the role of macrophages in cardiac homeostasis as well as after MI, with various current findings yet to be translated into actual clinical benefit.

## Data Availability

Not applicable.

## Abbreviations

MI: Myocardial infarction
AMI: Acute myocardial infarction
RTMs: Tissue-resident macrophages
CCR2: C–C motif chemokine receptor 2
HLA-DR: Human leukocyte antigen DR
Ly-6C: Lymphocyte antigen 6 complex
MHCII: Major histocompatibility complex II
TIMD4: T cell immunoglobulin and mucin domain containing 4
MMPs: Matrix metalloproteinases
CXCL: CXC motif chemokine ligand
TGF-β: Tumor growth factor-beta
TNC: Tenascin-C
LPS: Lipopolysaccharide
IRF: Interferon regulatory factor
TLR4: Toll like receptor 4
TNC: Tenascin-C
LGR: Leucine-rich repeat-containing G protein coupled receptor
AP-1: Activator protein-1
CREB: CAMP response element-binding protein
MST1/2: Mammalian STE20-like protein kinase 1/2
5-LOX: 5-Lipoxygenase
BLT1: Leukotriene B4 receptor 1
LTB4: Leukotriene B4
JAK: Janus kinase
STAT: Signal transducer and activator of transcription
Arg1: Arginase 1
Mrc1: Macrophage mannose receptor 1
Retnla: Resistin-like-α
Chi3l: Chitinase 3-like 3
PPAR: Peroxisome proliferator-activated receptors
C/EBP*β*: CCAAT enhancer binding protein beta
CMs: Cardiomyocytes
Nox4: NADPH oxidase 4
MSK: Mitogen- and stress-activated kinase
DUSP1: Dual-specificity protein phosphatase 1
MerTk: Myeloid-epithelial-reproductive tyrosine kinase
ICAM3: Intercellular adhesion molecule 3
TSP: Thrombospondin 1
Lgmn: Legumain
PARP2: Poly(ADP-ribose) polymerase 2
CMs: Cardiac myocytes
SIRPα: Signal regulatory protein alpha
LV: Left ventricle
TRPV4: Transient receptor potential vanilloid 4
BMDMs: Bone marrow-derived macrophages
ACE: Angiotensinogen converting enzyme
OSM: Oncostatin M
GPCMs: Gata6^+^ pericardial macrophages
DAMPs: Damage-related molecular patterns
NGAL: Neutrophil gelatinase-associated lipocalin
CX3CR: C-X3-C motif chemokine receptor
ANXA1: Annexin A1
VEGF: Vascular endothelial growth factor
IGF: Insulin-like growth factor
ECM: Extracellular matrix

## References

[1] Nielsen PH, Maeng M, Busk M, Mortensen LS, Kristensen SD, Nielsen TT, Andersen HR (2010). Primary angioplasty versus fibrinolysis in acute myocardial infarction: long-term follow-up in the Danish acute myocardial infarction 2 trial. Circulation.

[2] Keeley EC, Boura JA, Grines CL (2003). Primary angioplasty versus intravenous thrombolytic therapy for acute myocardial infarction: a quantitative review of 23 randomised trials. Lancet.

[3] Zijlstra F, Hoorntje JC, de Boer MJ, Reiffers S, Miedema K, Ottervanger JP, van ‘t Hof AW, Suryapranata H (1999). Long-term benefit of primary angioplasty as compared with thrombolytic therapy for acute myocardial infarction. N Engl J Med.

[4] Yellon DM, Hausenloy DJ (2007). Myocardial reperfusion injury. N Engl J Med.

[5] Cung TT, Morel O, Cayla G, Rioufol G, Garcia-Dorado D, Angoulvant D, Bonnefoy-Cudraz E, Guérin P, Elbaz M, Delarche N (2015). Cyclosporine before PCI in patients with acute myocardial infarction. N Engl J Med.

[6] Visan I (2018). Myocardial infarct inflammation. Nat Immunol.

[7] Nahrendorf M (2018). Myeloid cell contributions to cardiovascular health and disease. Nat Med.

[8] Jung K, Kim P, Leuschner F, Gorbatov R, Kim JK, Ueno T, Nahrendorf M, Yun SH (2013). Endoscopic time-lapse imaging of immune cells in infarcted mouse hearts. Circ Res.

[9] Lee WW, Marinelli B, van der Laan AM, Sena BF, Gorbatov R, Leuschner F, Dutta P, Iwamoto Y, Ueno T, Begieneman MP (2012). PET/MRI of inflammation in myocardial infarction. J Am Coll Cardiol.

[10] Nahrendorf M, Swirski FK, Aikawa E, Stangenberg L, Wurdinger T, Figueiredo JL, Libby P, Weissleder R, Pittet MJ (2007). The healing myocardium sequentially mobilizes two monocyte subsets with divergent and complementary functions. J Exp Med.

[11] Peet C, Ivetic A, Bromage DI, Shah AM (2020). Cardiac monocytes and macrophages after myocardial infarction. Cardiovasc Res.

[12] Ziegler-Heitbrock HW (1996). Heterogeneity of human blood monocytes: the CD14+ CD16+ subpopulation. Immunol Today.

[13] Ingersoll MA, Spanbroek R, Lottaz C, Gautier EL, Frankenberger M, Hoffmann R, Lang R, Haniffa M, Collin M, Tacke F (2010). Comparison of gene expression profiles between human and mouse monocyte subsets. Blood.

[14] Geissmann F, Jung S, Littman DR (2003). Blood monocytes consist of two principal subsets with distinct migratory properties. Immunity.

[15] Wynn TA, Chawla A, Pollard JW (2013). Macrophage biology in development, homeostasis and disease. Nature.

[16] Cossarizza A, Chang HD, Radbruch A, Acs A, Adam D, Adam-Klages S, Agace WW, Aghaeepour N, Akdis M, Allez M (2019). Guidelines for the use of flow cytometry and cell sorting in immunological studies (second edition). Eur J Immunol.

[17] Tariq M, Zhang JQ, Liang GK, He QJ, Ding L, Yang B (2017). Gefitinib inhibits M2-like polarization of tumor-associated macrophages in Lewis lung cancer by targeting the STAT6 signaling pathway. Acta Pharmacol Sin.

[18] Garaicoa-Pazmino C, Fretwurst T, Squarize CH, Berglundh T, Giannobile WV, Larsson L, Castilho RM (2019). Characterization of macrophage polarization in periodontal disease. J Clin Periodontol.

[19] Murray PJ (2017). Macrophage Polarization. Annu Rev Physiol.

[20] Gentek R, Molawi K, Sieweke MH (2014). Tissue macrophage identity and self-renewal. Immunol Rev.

[21] Davies LC, Jenkins SJ, Allen JE, Taylor PR (2013). Tissue-resident macrophages. Nat Immunol.

[22] Cassetta L, Pollard JW (2018). Targeting macrophages: therapeutic approaches in cancer. Nat Rev Drug Discov.

[23] Varol C, Mildner A, Jung S (2015). Macrophages: development and tissue specialization. Annu Rev Immunol.

[24] Taylor PR, Martinez-Pomares L, Stacey M, Lin HH, Brown GD, Gordon S (2005). Macrophage receptors and immune recognition. Annu Rev Immunol.

[25] Hesketh M, Sahin KB, West ZE, Murray RZ (2017). Macrophage phenotypes regulate scar formation and chronic wound healing. Int J Mol Sci.

[26] Passlick B, Flieger D, Ziegler-Heitbrock HW (1989). Identification and characterization of a novel monocyte subpopulation in human peripheral blood. Blood.

[27] Honold L, Nahrendorf M (2018). Resident and monocyte-derived macrophages in cardiovascular disease. Circ Res.

[28] Sica A, Erreni M, Allavena P, Porta C (2015). Macrophage polarization in pathology. Cell Mol Life Sci.

[29] Hume DA, MacDonald KP (2012). Therapeutic applications of macrophage colony-stimulating factor-1 (CSF-1) and antagonists of CSF-1 receptor (CSF-1R) signaling. Blood.

[30] Wong NR, Mohan J, Kopecky BJ, Guo S, Du L, Leid J, Feng G, Lokshina I, Dmytrenko O, Luehmann H (2021). Resident cardiac macrophages mediate adaptive myocardial remodeling. Immunity.

[31] Culemann S, Grüneboom A, Nicolás-Ávila J, Weidner D, Lämmle KF, Rothe T, Quintana JA, Kirchner P, Krljanac B, Eberhardt M (2019). Locally renewing resident synovial macrophages provide a protective barrier for the joint. Nature.

[32] Lavine KJ, Epelman S, Uchida K, Weber KJ, Nichols CG, Schilling JD, Ornitz DM, Randolph GJ, Mann DL (2014). Distinct macrophage lineages contribute to disparate patterns of cardiac recovery and remodeling in the neonatal and adult heart. Proc Natl Acad Sci U S A.

[33] Leid J, Carrelha J, Boukarabila H, Epelman S, Jacobsen SE, Lavine KJ (2016). Primitive embryonic macrophages are required for coronary development and maturation. Circ Res.

[34] Barth MW, Hendrzak JA, Melnicoff MJ, Morahan PS (1995). Review of the macrophage disappearance reaction. J Leukoc Biol.

[35] Davies LC, Rosas M, Jenkins SJ, Liao CT, Scurr MJ, Brombacher F, Fraser DJ, Allen JE, Jones SA, Taylor PR (1886). Distinct bone marrow-derived and tissue-resident macrophage lineages proliferate at key stages during inflammation. Nat Commun.

[36] Okabe Y, Medzhitov R (2016). Tissue biology perspective on macrophages. Nat Immunol.

[37] Blériot C, Chakarov S, Ginhoux F (2020). Determinants of resident tissue macrophage identity and function. Immunity.

[38] Hoeffel G, Chen J, Lavin Y, Low D, Almeida FF, See P, Beaudin AE, Lum J, Low I, Forsberg EC (2015). C-Myb(+) erythro-myeloid progenitor-derived fetal monocytes give rise to adult tissue-resident macrophages. Immunity.

[39] Bajpai G, Schneider C, Wong N, Bredemeyer A, Hulsmans M, Nahrendorf M, Epelman S, Kreisel D, Liu Y, Itoh A (2018). The human heart contains distinct macrophage subsets with divergent origins and functions. Nat Med.

[40] Pinto AR, Paolicelli R, Salimova E, Gospocic J, Slonimsky E, Bilbao-Cortes D, Godwin JW, Rosenthal NA (2012). An abundant tissue macrophage population in the adult murine heart with a distinct alternatively-activated macrophage profile. PLoS ONE.

[41] Bajpai G, Bredemeyer A, Li W, Zaitsev K, Koenig AL, Lokshina I, Mohan J, Ivey B, Hsiao HM, Weinheimer C (2019). Tissue resident CCR2- and CCR2+ cardiac macrophages differentially orchestrate monocyte recruitment and fate specification following myocardial injury. Circ Res.

[42] Chakarov S, Lim HY, Tan L, Lim SY, See P, Lum J, Zhang XM, Foo S, Nakamizo S, Duan K (2019). Two distinct interstitial macrophage populations coexist across tissues in specific subtissular niches. Science.

[43] Heidt T, Courties G, Dutta P, Sager HB, Sebas M, Iwamoto Y, Sun Y, Da Silva N, Panizzi P, van der Laan AM (2014). Differential contribution of monocytes to heart macrophages in steady-state and after myocardial infarction. Circ Res.

[44] Rizzo G, Vafadarnejad E, Arampatzi P, Silvestre J-S, Zernecke A, Saliba A-E, Cochain C. Single-cell transcriptomic profiling maps monocyte/macrophage transitions after myocardial infarction in mice. BioRxiv. 2020.

[45] Ryabov V, Gombozhapova A, Rogovskaya Y, Kzhyshkowska J, Rebenkova M, Karpov R (2018). Cardiac CD68+ and stabilin-1+ macrophages in wound healing following myocardial infarction: From experiment to clinic. Immunobiology.

[46] Dick SA, Macklin JA, Nejat S, Momen A, Clemente-Casares X, Althagafi MG, Chen J, Kantores C, Hosseinzadeh S, Aronoff L (2019). Self-renewing resident cardiac macrophages limit adverse remodeling following myocardial infarction. Nat Immunol.

[47] Sager HB, Hulsmans M, Lavine KJ, Moreira MB, Heidt T, Courties G, Sun Y, Iwamoto Y, Tricot B, Khan OF (2016). Proliferation and recruitment contribute to myocardial macrophage expansion in chronic heart failure. Circ Res.

[48] Ma Y, Iyer RP, Jung M, Czubryt MP, Lindsey ML (2017). Cardiac Fibroblast activation post-myocardial infarction: current knowledge gaps. Trends Pharmacol Sci.

[49] Dassanayaka S, Jones SP (2015). Recent developments in heart failure. Circ Res.

[50] Ma Y, Mouton AJ, Lindsey ML (2018). Cardiac macrophage biology in the steady-state heart, the aging heart, and following myocardial infarction. Transl Res.

[51] Lindsey ML, Saucerman JJ, DeLeon-Pennell KY (2016). Knowledge gaps to understanding cardiac macrophage polarization following myocardial infarction. Biochim Biophys Acta.

[52] Tourki B, Halade G (2017). Leukocyte diversity in resolving and nonresolving mechanisms of cardiac remodeling. Faseb j.

[53] Frangogiannis NG (2012). Regulation of the inflammatory response in cardiac repair. Circ Res.

[54] Lawrence T, Natoli G (2011). Transcriptional regulation of macrophage polarization: enabling diversity with identity. Nat Rev Immunol.

[55] Gordon S, Taylor PR (2005). Monocyte and macrophage heterogeneity. Nat Rev Immunol.

[56] Hanna RN, Carlin LM, Hubbeling HG, Nackiewicz D, Green AM, Punt JA, Geissmann F, Hedrick CC (2011). The transcription factor NR4A1 (Nur77) controls bone marrow differentiation and the survival of Ly6C- monocytes. Nat Immunol.

[57] Toshchakov V, Jones BW, Perera PY, Thomas K, Cody MJ, Zhang S, Williams BR, Major J, Hamilton TA, Fenton MJ, Vogel SN (2002). TLR4, but not TLR2, mediates IFN-beta-induced STAT1alpha/beta-dependent gene expression in macrophages. Nat Immunol.

[58] Kimura T, Tajiri K, Sato A, Sakai S, Wang Z, Yoshida T, Uede T, Hiroe M, Aonuma K, Ieda M, Imanaka-Yoshida K (2019). Tenascin-C accelerates adverse ventricular remodelling after myocardial infarction by modulating macrophage polarization. Cardiovasc Res.

[59] Huang CK, Dai D, Xie H, Zhu Z, Hu J, Su M, Liu M, Lu L, Shen W, Ning G (2020). Lgr4 governs a pro-inflammatory program in macrophages to antagonize post-infarction cardiac repair. Circ Res.

[60] Liu M, Yan M, He J, Lv H, Chen Z, Peng L, Cai W, Yao F, Chen C, Shi L (2021). Macrophage MST1/2 disruption impairs post-infarction cardiac repair via LTB4. Circ Res.

[61] Martinez FO, Helming L, Gordon S (2009). Alternative activation of macrophages: an immunologic functional perspective. Annu Rev Immunol.

[62] Szanto A, Balint BL, Nagy ZS, Barta E, Dezso B, Pap A, Szeles L, Poliska S, Oros M, Evans RM (2010). STAT6 transcription factor is a facilitator of the nuclear receptor PPARγ-regulated gene expression in macrophages and dendritic cells. Immunity.

[63] Chen B, Huang S, Su Y, Wu YJ, Hanna A, Brickshawana A, Graff J, Frangogiannis NG (2019). Macrophage Smad3 protects the infarcted heart, stimulating phagocytosis and regulating inflammation. Circ Res.

[64] Zlatanova I, Pinto C, Bonnin P, Mathieu JRR, Bakker W, Vilar J, Lemitre M, Voehringer D, Vaulont S, Peyssonnaux C, Silvestre JS (2019). Iron regulator hepcidin impairs macrophage-dependent cardiac repair after injury. Circulation.

[65] Ma Y, Halade GV, Zhang J, Ramirez TA, Levin D, Voorhees A, Jin YF, Han HC, Manicone AM, Lindsey ML (2013). Matrix metalloproteinase-28 deletion exacerbates cardiac dysfunction and rupture after myocardial infarction in mice by inhibiting M2 macrophage activation. Circ Res.

[66] Mongue-Din H, Patel AS, Looi YH, Grieve DJ, Anilkumar N, Sirker A, Dong X, Brewer AC, Zhang M, Smith A, Shah AM (2017). NADPH oxidase-4 driven cardiac macrophage polarization protects against myocardial infarction-induced remodeling. JACC Basic Transl Sci.

[67] Ruffell D, Mourkioti F, Gambardella A, Kirstetter P, Lopez RG, Rosenthal N, Nerlov C (2009). A CREB-C/EBPbeta cascade induces M2 macrophage-specific gene expression and promotes muscle injury repair. Proc Natl Acad Sci U S A.

[68] Kim C, Wilcox-Adelman S, Sano Y, Tang WJ, Collier RJ, Park JM (2008). Antiinflammatory cAMP signaling and cell migration genes co-opted by the anthrax bacillus. Proc Natl Acad Sci U S A.

[69] Ananieva O, Darragh J, Johansen C, Carr JM, McIlrath J, Park JM, Wingate A, Monk CE, Toth R, Santos SG (2008). The kinases MSK1 and MSK2 act as negative regulators of Toll-like receptor signaling. Nat Immunol.

[70] Otaka N, Shibata R, Ohashi K, Uemura Y, Kambara T, Enomoto T, Ogawa H, Ito M, Kawanishi H, Maruyama S (2018). Myonectin is an exercise-induced myokine that protects the heart from ischemia-reperfusion injury. Circ Res.

[71] Marinković G, Grauen Larsen H, Yndigegn T, Szabo IA, Mares RG, de Camp L, Weiland M, Tomas L, Goncalves I, Nilsson J (2019). Inhibition of pro-inflammatory myeloid cell responses by short-term S100A9 blockade improves cardiac function after myocardial infarction. Eur Heart J.

[72] Marinković G, Koenis DS, de Camp L, Jablonowski R, Graber N, de Waard V, de Vries CJ, Goncalves I, Nilsson J, Jovinge S, Schiopu A (2020). S100A9 links inflammation and repair in myocardial infarction. Circ Res.

[73] Yang K, Xiao Q, Niu M, Pan X, Zhu X (2022). Exosomes in atherosclerosis: convergence on macrophages. Int J Biol Sci.

[74] Xia Y, Rao L, Yao H, Wang Z, Ning P, Chen X (2020). Engineering Macrophages for cancer immunotherapy and drug delivery. Adv Mater.

[75] Lauber K, Blumenthal SG, Waibel M, Wesselborg S (2004). Clearance of apoptotic cells: getting rid of the corpses. Mol Cell.

[76] Gregory C (2009). Cell biology: sent by the scent of death. Nature.

[77] Elliott MR, Chekeni FB, Trampont PC, Lazarowski ER, Kadl A, Walk SF, Park D, Woodson RI, Ostankovich M, Sharma P (2009). Nucleotides released by apoptotic cells act as a find-me signal to promote phagocytic clearance. Nature.

[78] Lauber K, Bohn E, Kröber SM, Xiao YJ, Blumenthal SG, Lindemann RK, Marini P, Wiedig C, Zobywalski A, Baksh S (2003). Apoptotic cells induce migration of phagocytes via caspase-3-mediated release of a lipid attraction signal. Cell.

[79] Gude DR, Alvarez SE, Paugh SW, Mitra P, Yu J, Griffiths R, Barbour SE, Milstien S, Spiegel S (2008). Apoptosis induces expression of sphingosine kinase 1 to release sphingosine-1-phosphate as a "come-and-get-me" signal. Faseb J.

[80] Moffatt OD, Devitt A, Bell ED, Simmons DL, Gregory CD (1999). Macrophage recognition of ICAM-3 on apoptotic leukocytes. J Immunol.

[81] Savill J, Hogg N, Ren Y, Haslett C (1992). Thrombospondin cooperates with CD36 and the vitronectin receptor in macrophage recognition of neutrophils undergoing apoptosis. J Clin Invest.

[82] Grimsley C, Ravichandran KS (2003). Cues for apoptotic cell engulfment: eat-me, don't eat-me and come-get-me signals. Trends Cell Biol.

[83] Elliott MR, Ravichandran KS (2016). The dynamics of apoptotic cell clearance. Dev Cell.

[84] Poon IK, Lucas CD, Rossi AG, Ravichandran KS (2014). Apoptotic cell clearance: basic biology and therapeutic potential. Nat Rev Immunol.

[85] Kourtzelis I, Hajishengallis G, Chavakis T (2020). Phagocytosis of apoptotic cells in resolution of inflammation. Front Immunol.

[86] Ungewickell EJ, Hinrichsen L (2007). Endocytosis: clathrin-mediated membrane budding. Curr Opin Cell Biol.

[87] Kinchen JM, Ravichandran KS (2008). Phagosome maturation: going through the acid test. Nat Rev Mol Cell Biol.

[88] Ravichandran KS (2010). Find-me and eat-me signals in apoptotic cell clearance: progress and conundrums. J Exp Med.

[89] Boada-Romero E, Martinez J, Heckmann BL, Green DR (2020). The clearance of dead cells by efferocytosis. Nat Rev Mol Cell Biol.

[90] Savill J, Dransfield I, Gregory C, Haslett C (2002). A blast from the past: clearance of apoptotic cells regulates immune responses. Nat Rev Immunol.

[91] Nicolás-Ávila JA, Lechuga-Vieco AV, Esteban-Martínez L, Sánchez-Díaz M, Díaz-García E, Santiago DJ, Rubio-Ponce A, Li JL, Balachander A, Quintana JA (2020). A network of macrophages supports mitochondrial homeostasis in the heart. Cell.

[92] Jia D, Chen S, Bai P, Luo C, Liu J, Sun A, Ge J (2022). Cardiac resident macrophage-derived Legumain improves cardiac repair by promoting clearance and degradation of apoptotic cardiomyocytes after myocardial infarction. Circulation.

[93] Pinto AR, Ilinykh A, Ivey MJ, Kuwabara JT, D'Antoni ML, Debuque R, Chandran A, Wang L, Arora K, Rosenthal NA, Tallquist MD (2016). Revisiting cardiac cellular composition. Circ Res.

[94] Frangogiannis NG (2014). The immune system and the remodeling infarcted heart: cell biological insights and therapeutic opportunities. J Cardiovasc Pharmacol.

[95] Zhang S, Yeap XY, DeBerge M, Naresh NK, Wang K, Jiang Z, Wilcox JE, White SM, Morrow JP, Burridge PW (2017). Acute CD47 blockade during ischemic myocardial reperfusion enhances phagocytosis-associated cardiac repair. JACC Basic Transl Sci.

[96] Elliott MR, Ravichandran KS (2010). Clearance of apoptotic cells: implications in health and disease. J Cell Biol.

[97] Zhang S, Yeap XY, Grigoryeva L, Dehn S, DeBerge M, Tye M, Rostlund E, Schrijvers D, Zhang ZJ, Sumagin R (2015). Cardiomyocytes induce macrophage receptor shedding to suppress phagocytosis. J Mol Cell Cardiol.

[98] Zagórska A, Través PG, Lew ED, Dransfield I, Lemke G (2014). Diversification of TAM receptor tyrosine kinase function. Nat Immunol.

[99] Cai B, Thorp EB, Doran AC, Sansbury BE, Daemen MJ, Dorweiler B, Spite M, Fredman G, Tabas I (2017). MerTK receptor cleavage promotes plaque necrosis and defective resolution in atherosclerosis. J Clin Invest.

[100] Cai B, Thorp EB, Doran AC, Subramanian M, Sansbury BE, Lin CS, Spite M, Fredman G, Tabas I (2016). MerTK cleavage limits proresolving mediator biosynthesis and exacerbates tissue inflammation. Proc Natl Acad Sci U S A.

[101] Han C, Nie Y, Lian H, Liu R, He F, Huang H, Hu S (2015). Acute inflammation stimulates a regenerative response in the neonatal mouse heart. Cell Res.

[102] Bassat E, Mutlak YE, Genzelinakh A, Shadrin IY, Baruch Umansky K, Yifa O, Kain D, Rajchman D, Leach J, Riabov Bassat D (2017). The extracellular matrix protein agrin promotes heart regeneration in mice. Nature.

[103] Xin M, Kim Y, Sutherland LB, Murakami M, Qi X, McAnally J, Porrello ER, Mahmoud AI, Tan W, Shelton JM (2013). Hippo pathway effector Yap promotes cardiac regeneration. Proc Natl Acad Sci U S A.

[104] Li Y, Feng J, Song S, Li H, Yang H, Zhou B, Li Y, Yue Z, Lian H, Liu L (2020). gp130 controls cardiomyocyte proliferation and heart regeneration. Circulation.

[105] Hu S, Gao Y, Gao R, Wang Y, Qu Y, Yang J, Wei X, Zhang F, Ge J (2022). The selective STING inhibitor H-151 preserves myocardial function and ameliorates cardiac fibrosis in murine myocardial infarction. Int Immunopharmacol.

[106] Yan M, Yang Y, Zhou Y, Yu C, Li R, Gong W, Zheng J (2021). Interleukin-7 aggravates myocardial ischaemia/reperfusion injury by regulating macrophage infiltration and polarization. J Cell Mol Med.

[107] Prabhu SD, Frangogiannis NG (2016). The biological basis for cardiac repair after myocardial infarction: from inflammation to fibrosis. Circ Res.

[108] Weber KT, Sun Y, Bhattacharya SK, Ahokas RA, Gerling IC (2013). Myofibroblast-mediated mechanisms of pathological remodelling of the heart. Nat Rev Cardiol.

[109] Shinde AV, Frangogiannis NG (2014). Fibroblasts in myocardial infarction: a role in inflammation and repair. J Mol Cell Cardiol.

[110] van den Borne SW, Diez J, Blankesteijn WM, Verjans J, Hofstra L, Narula J (2010). Myocardial remodeling after infarction: the role of myofibroblasts. Nat Rev Cardiol.

[111] Mantovani A, Biswas SK, Galdiero MR, Sica A, Locati M (2013). Macrophage plasticity and polarization in tissue repair and remodelling. J Pathol.

[112] Martínez-Martínez E, Calvier L, Fernández-Celis A, Rousseau E, Jurado-López R, Rossoni LV, Jaisser F, Zannad F, Rossignol P, Cachofeiro V, López-Andrés N (2015). Galectin-3 blockade inhibits cardiac inflammation and fibrosis in experimental hyperaldosteronism and hypertension. Hypertension.

[113] Frangogiannis NG (2019). Cardiac fibrosis: Cell biological mechanisms, molecular pathways and therapeutic opportunities. Mol Aspects Med.

[114] Haider N, Boscá L, Zandbergen HR, Kovacic JC, Narula N, González-Ramos S, Fernandez-Velasco M, Agrawal S, Paz-García M, Gupta S (2019). Transition of macrophages to fibroblast-like cells in healing myocardial infarction. J Am Coll Cardiol.

[115] Wang Y, Li C, Zhao R, Qiu Z, Shen C, Wang Z, Liu W, Zhang W, Ge J, Shi B (2021). CircUbe3a from M2 macrophage-derived small extracellular vesicles mediates myocardial fibrosis after acute myocardial infarction. Theranostics.

[116] Abe H, Takeda N, Isagawa T, Semba H, Nishimura S, Morioka MS, Nakagama Y, Sato T, Soma K, Koyama K (2019). Macrophage hypoxia signaling regulates cardiac fibrosis via Oncostatin M. Nat Commun.

[117] Wang C, Zhang C, Liu L, Xi A, Chen B, Li Y, Du J (2017). Macrophage-derived mir-155-containing exosomes suppress fibroblast proliferation and promote fibroblast inflammation during cardiac injury. Mol Ther.

[118] Deniset JF, Belke D, Lee W-Y, Jorch SK, Deppermann C, Hassanabad AF, Turnbull JD, Teng G, Rozich I, Hudspeth K (2019). Gata6+ pericardial cavity macrophages relocate to the injured heart and prevent cardiac fibrosis. Immunity.

[119] Jin H, Liu K, Huang X, Huo H, Mou J, Qiao Z, He B, Zhou B (2022). Genetic lineage tracing of pericardial cavity macrophages in the injured heart. Circ Res.

[120] Gori AM, Cesari F, Marcucci R, Giusti B, Paniccia R, Antonucci E, Gensini GF, Abbate R (2009). The balance between pro- and anti-inflammatory cytokines is associated with platelet aggregability in acute coronary syndrome patients. Atherosclerosis.

[121] Kologrivova I, Shtatolkina M, Suslova T, Ryabov V (2021). Cells of the immune system in cardiac remodeling: main players in resolution of inflammation and repair after myocardial infarction. Front Immunol.

[122] Wei X, Zou S, Xie Z, Wang Z, Huang N, Cen Z, Hao Y, Zhang C, Chen Z, Zhao F (2021). EDIL3 deficiency ameliorates adverse cardiac remodeling by neutrophil extracellular traps (NET)-mediated macrophage polarization. Cardiovasc Res..

[123] Kolaczkowska E, Kubes P (2013). Neutrophil recruitment and function in health and inflammation. Nat Rev Immunol.

[124] Döring Y, Drechsler M, Soehnlein O, Weber C (2015). Neutrophils in atherosclerosis: from mice to man. Arterioscler Thromb Vasc Biol.

[125] Horckmans M, Ring L, Duchene J, Santovito D, Schloss MJ, Drechsler M, Weber C, Soehnlein O, Steffens S (2017). Neutrophils orchestrate post-myocardial infarction healing by polarizing macrophages towards a reparative phenotype. Eur Heart J.

[126] Kubota A, Suto A, Suzuki K, Kobayashi Y, Nakajima H (2019). Matrix metalloproteinase-12 produced by Ly6C(low) macrophages prolongs the survival after myocardial infarction by preventing neutrophil influx. J Mol Cell Cardiol.

[127] Daseke MJ, Tenkorang-Impraim MAA, Ma Y, Chalise U, Konfrst SR, Garrett MR, DeLeon-Pennell KY, Lindsey ML (2020). Exogenous IL-4 shuts off pro-inflammation in neutrophils while stimulating anti-inflammation in macrophages to induce neutrophil phagocytosis following myocardial infarction. J Mol Cell Cardiol.

[128] Hofmann U, Frantz S (2016). Role of T-cells in myocardial infarction. Eur Heart J.

[129] Ramos G, Hofmann U, Frantz S (2016). Myocardial fibrosis seen through the lenses of T-cell biology. J Mol Cell Cardiol.

[130] Jia D, Jiang H, Weng X, Wu J, Bai P, Yang W, Wang Z, Hu K, Sun A, Ge J (2019). Interleukin-35 promotes macrophage survival and improves wound healing after myocardial infarction in mice. Circ Res.

[131] Farache Trajano L, Smart N (2021). Immunomodulation for optimal cardiac regeneration: insights from comparative analyses. NPJ Regen Med.

[132] Toor IS, Rückerl D, Mair I, Ainsworth R, Meloni M, Spiroski AM, Benezech C, Felton JM, Thomson A, Caporali A (2020). Eosinophil deficiency promotes aberrant repair and adverse remodeling following acute myocardial infarction. JACC Basic Transl Sci.

[133] Xu JY, Xiong YY, Tang RJ, Jiang WY, Ning Y, Gong ZT, Huang PS, Chen GH, Xu J, Wu CX (2021). Interleukin-5-induced eosinophil population improves cardiac function after myocardial infarction. Cardiovasc Res..

[134] Sicklinger F, Meyer IS, Li X, Radtke D, Dicks S, Kornadt MP, Mertens C, Meier JK, Lavine KJ, Zhang Y (2021). Basophils balance healing after myocardial infarction via IL-4/IL-13. J Clin Invest.

[135] Fernández-Ruiz I (2021). Basophils promote healing after MI. Nat Rev Cardiol.

[136] Prabhu SD (2021). Healing and repair after myocardial infarction: the forgotten but resurgent basophil. J Clin Invest.

[137] Liu S, Chen J, Shi J, Zhou W, Wang L, Fang W, Zhong Y, Chen X, Chen Y, Sabri A, Liu S (2020). M1-like macrophage-derived exosomes suppress angiogenesis and exacerbate cardiac dysfunction in a myocardial infarction microenvironment. Basic Res Cardiol.

[138] Deveza L, Choi J, Yang F (2012). Therapeutic angiogenesis for treating cardiovascular diseases. Theranostics.

[139] Hausenloy DJ, Chilian W, Crea F, Davidson SM, Ferdinandy P, Garcia-Dorado D, van Royen N, Schulz R, Heusch G (2019). The coronary circulation in acute myocardial ischaemia/reperfusion injury: a target for cardioprotection. Cardiovasc Res.

[140] Ferraro B, Leoni G, Hinkel R, Ormanns S, Paulin N, Ortega-Gomez A, Viola JR, de Jong R, Bongiovanni D, Bozoglu T (2019). Pro-angiogenic macrophage phenotype to promote myocardial repair. J Am Coll Cardiol.

[141] Zou J, Fei Q, Xiao H, Wang H, Liu K, Liu M, Zhang H, Xiao X, Wang K, Wang N (2019). VEGF-A promotes angiogenesis after acute myocardial infarction through increasing ROS production and enhancing ER stress-mediated autophagy. J Cell Physiol.

[142] Hu C, Wu Z, Huang Z, Hao X, Wang S, Deng J, Yin Y, Tan C (2021). Nox2 impairs VEGF-A-induced angiogenesis in placenta via mitochondrial ROS-STAT3 pathway. Redox Biol.

[143] Pignata P, Apicella I, Cicatiello V, Puglisi C, Magliacane Trotta S, Sanges R, Tarallo V, De Falco S (2021). Prolyl 3-Hydroxylase 2 Is a Molecular Player of Angiogenesis. Int J Mol Sci.

[144] Page-McCaw A, Ewald AJ, Werb Z (2007). Matrix metalloproteinases and the regulation of tissue remodelling. Nat Rev Mol Cell Biol.

[145] Sato H, Takino T, Okada Y, Cao J, Shinagawa A, Yamamoto E, Seiki M (1994). A matrix metalloproteinase expressed on the surface of invasive tumour cells. Nature.

[146] Alonso-Herranz L, Sahún-Español Á, Paredes A, Gonzalo P, Gkontra P, Núñez V, Clemente C, Cedenilla M, Villalba-Orero M, Inserte J (2020). Macrophages promote endothelial-to-mesenchymal transition via MT1-MMP/TGFβ1 after myocardial infarction. Elife.

[147] Lee SJ, Lee CK, Kang S, Park I, Kim YH, Kim SK, Hong SP, Bae H, He Y, Kubota Y, Koh GY (2018). Angiopoietin-2 exacerbates cardiac hypoxia and inflammation after myocardial infarction. J Clin Invest.

[148] Kuang Y, Li X, Liu X, Wei L, Chen X, Liu J, Zhuang T, Pi J, Wang Y, Zhu C (2021). Vascular endothelial S1pr1 ameliorates adverse cardiac remodelling via stimulating reparative macrophage proliferation after myocardial infarction. Cardiovasc Res.

[149] Reboll MR, Klede S, Taft MH, Cai CL, Field LJ, Lavine KJ, Koenig AL, Fleischauer J, Meyer J, Schambach A (2022). Meteorin-like promotes heart repair through endothelial KIT receptor tyrosine kinase. Science.

[150] Vieira JM, Norman S, Villa Del Campo C, Cahill TJ, Barnette DN, Gunadasa-Rohling M, Johnson LA, Greaves DR, Carr CA, Jackson DG, Riley PR (2018). The cardiac lymphatic system stimulates resolution of inflammation following myocardial infarction. J Clin Invest.

[151] Klotz L, Norman S, Vieira JM, Masters M, Rohling M, Dubé KN, Bollini S, Matsuzaki F, Carr CA, Riley PR (2015). Cardiac lymphatics are heterogeneous in origin and respond to injury. Nature.

[152] Kataru RP, Jung K, Jang C, Yang H, Schwendener RA, Baik JE, Han SH, Alitalo K, Koh GY (2009). Critical role of CD11b+ macrophages and VEGF in inflammatory lymphangiogenesis, antigen clearance, and inflammation resolution. Blood.

[153] Glinton KE, Ma W, Lantz C, Grigoryeva LS, DeBerge M, Liu X, Febbraio M, Kahn M, Oliver G, Thorp EB (2022). Macrophage-produced VEGFC is induced by efferocytosis to ameliorate cardiac injury and inflammation. J Clin Invest.

[154] Qiu N, Wang G, Wang J, Zhou Q, Guo M, Wang Y, Hu X, Zhou H, Bai R, You M (2021). Tumor-associated macrophage and tumor-cell dually transfecting polyplexes for efficient interleukin-12 cancer gene therapy. Adv Mater.

[155] Chen Q, Gao M, Li Z, Xiao Y, Bai X, Boakye-Yiadom KO, Xu X, Zhang XQ (2020). Biodegradable nanoparticles decorated with different carbohydrates for efficient macrophage-targeted gene therapy. J Control Release.

[156] Haney MJ, Klyachko NL, Zhao Y, Gupta R, Plotnikova EG, He Z, Patel T, Piroyan A, Sokolsky M, Kabanov AV, Batrakova EV (2015). Exosomes as drug delivery vehicles for Parkinson's disease therapy. J Control Release.

[157] Yang T, Hu Y, Miao J, Chen J, Liu J, Cheng Y, Gao X (2022). A BRD4 PROTAC nanodrug for glioma therapy via the intervention of tumor cells proliferation, apoptosis and M2 macrophages polarization. Acta Pharm Sin B.

